# Supplementary material for: Predicting visual function by interpreting a neuronal wiring diagram
Source: Nature. 2024 Oct 2;634(8032):113–23. doi: 10.1038/s41586-024-07953-5 (PMC11446822; doi:10.1038/s41586-024-07953-5)
Supplement: Supplementary file 6 — CRF and ERF predictions for individual TmY4 and TmY9 cells. Analogous to Supplementary Data 3, but for TmY target types. Shown are the top four monosynaptic pathways, the strongest pathway passing through each of the top ten intermediary types (ranking from Extended Data Fig. 7), and the trisynaptic pathway Tm1–TmY–Dm3–TmY (see the section entitled Prediction of spatial normalization). [file 41586_2024_7953_MOESM6_ESM.zip › DataS4/TmY9qΓèÑ/720575940631093199.pdf]

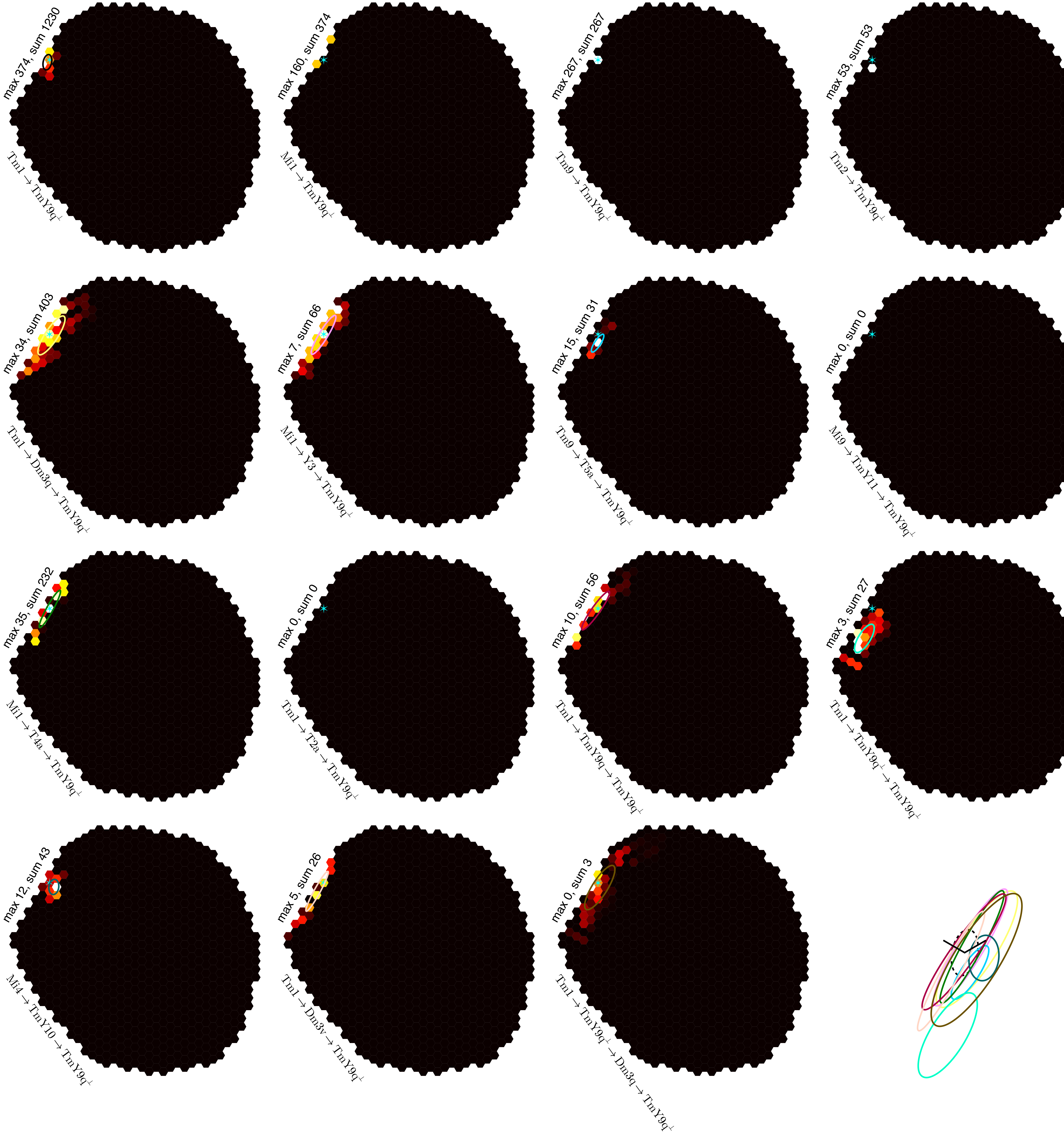

max 374, sum 1230
Tm1 → TmY9q⊥
max 160, sum 374
Mi1 → TmY9q⊥
max 267, sum 267
Tm9 → TmY9q⊥
max 53, sum 53
Tm2 → TmY9q⊥
max 34, sum 403
Tm1 → Dm3q → TmY9q⊥
max 7, sum 66
Mi1 → Y3 → TmY9q⊥
max 15, sum 31
Tm9 → T5a → TmY9q⊥
max 0, sum 0
Mi9 → TmY11 → TmY9q⊥
max 35, sum 232
Mi1 → T4a → TmY9q⊥
max 0, sum 0
Tm1 → T2a → TmY9q⊥
max 10, sum 56
Tm1 → TmY9q → TmY9q⊥
max 3, sum 27
Tm1 → TmY9q⊥ → TmY9q⊥
max 12, sum 43
Mi4 → TmY10 → TmY9q⊥
max 5, sum 26
Tm1 → Dm3v → TmY9q⊥
max 0, sum 3
Tm1 → TmY9q⊥ → Dm3q → TmY9q⊥
